# Radiotherapy for Adrenal Metastases from Hepatocellular Carcinoma: A 20-Year Bi-Institutional Experience

**DOI:** 10.3390/curroncol33060328

**Published:** 2026-06-01

**Authors:** Jeongshim Lee, Myungsoo Kim

**Affiliations:** 1 Department of Radiation Oncology, Inha University Hospital, Inha University School of Medicine, Incheon 22332, Republic of Korea; jshimlee@inha.ac.kr; 2 Department of Radiation Oncology, Incheon St. Mary’s Hospital, College of Medicine, The Catholic University of Korea, Seoul 06591, Republic of Korea

**Keywords:** hepatocellular carcinoma, adrenal metastasis, radiotherapy, stereotactic body radiotherapy, MR-guided radiotherapy, local control, overall survival

## Abstract

Background: To evaluate tumor response, local control (LC), survival, and toxicity after radiotherapy for adrenal metastases from hepatocellular carcinoma (HCC), we conducted a retrospective, bi-institutional study spanning two decades. Methods: Twenty patients received radiotherapy for adrenal metastases (2005–2025). Techniques included three-dimensional conformal radiotherapy, intensity-modulated radiotherapy, stereotactic body radiotherapy, and MR-guided radiotherapy. Tumor response was assessed using Response Evaluation Criteria in Solid Tumors version 1.1. LC, overall survival (OS), and progression-free survival (PFS) were estimated using Kaplan–Meier; the association between biologically effective dose (BED_10_) and local recurrence was assessed using Fisher’s exact test. Results: The objective response rate was 55.0%, and the disease control rate was 95.0%. The 6- and 12-month adrenal LC rates were 94.4% and 87.2%; local recurrence occurred in three patients (15.0%). No local recurrence was observed at BED_10_ ≥ 75 Gy (0/8) versus 25.0% (3/12) at BED_10_ < 75 Gy. However, this difference did not reach statistical significance (*p* = 0.242, Fisher’s exact test) and should be interpreted as exploratory and hypothesis-generating given the limited sample size. Median OS was 13.4 months, and median PFS was 6.3 months. Systemic progression was the predominant failure pattern. No grade ≥3 radiotherapy-related toxicity was observed. Conclusions: Radiotherapy achieved favorable LC with acceptable toxicity across a 20-year bi-institutional experience, supporting its role as an effective local treatment modality for adrenal metastases from HCC.

## Simple Summary

Adrenal metastases are a serious complication of liver cancer that can cause hormonal problems and worsen prognosis, yet optimal treatment remains uncertain. Radiotherapy is one of the few local treatment options available, particularly for patients who are not surgical candidates, but long-term evidence specific to liver cancer remains scarce. In this study, we examined outcomes of radiotherapy in patients with adrenal metastases from liver cancer treated at two Korean hospitals over 20 years. We found that radiotherapy effectively controlled the local tumor in most patients, with a 12-month local control rate of 87.2% and a median survival of 13.4 months. More than half showed measurable tumor shrinkage, and no severe treatment-related side effects were observed. Higher radiation doses appeared to be associated with better local control, although this finding did not reach statistical significance and should be considered exploratory. Nevertheless, cancer spread to other sites remained the main cause of treatment failure, underscoring the need to combine radiotherapy with modern drug therapies such as immunotherapy.

## 1. Introduction

Hepatocellular carcinoma (HCC) ranks sixth in global cancer incidence and third in cancer-related mortality, with an estimated 866,000 new cases and 759,000 deaths reported in 2022 [1]. Despite substantial advances in surveillance, locoregional therapies, and systemic treatments, the prognosis of patients with advanced HCC remains poor. Over time, therapeutic advances have prolonged survival; consequently, extrahepatic metastases—once rarely encountered—are now observed with increasing frequency as patients live longer. Extrahepatic dissemination occurs in approximately 13.5–42% of patients with advanced HCC, most commonly involving the lungs (53.8–74.5%), followed by lymph nodes (33.8%), bone (24.8–38.5%), and the adrenal glands (8.0–19.1%) [2,3]. Among these sites, the adrenal glands constitute the fourth most common site of extrahepatic metastasis from HCC; however, adrenal metastases carry disproportionate clinical significance because of their association with hormonal complications, local symptoms, and poor prognosis [3].

Various local and systemic treatment modalities have been explored for adrenal metastases from HCC. Local options include surgical adrenalectomy, minimally invasive ablative procedures such as radiofrequency ablation and microwave ablation, and radiotherapy [4]. Among local therapies, adrenalectomy has demonstrated relatively favorable outcomes in carefully selected patients with well-controlled intrahepatic disease and adequate hepatic reserve; however, it is applicable to only a minority of patients [4]. Systemic options include cytotoxic chemotherapy, tyrosine kinase inhibitors (TKIs), and immune checkpoint inhibitors (ICIs). For patients who are not suitable candidates for surgery or ablation, radiotherapy has been the most extensively studied local ablative modality, with a median overall survival (OS) of approximately 10–15 months reported in HCC-specific series [5,6]. Nevertheless, no consensus regarding the optimal treatment strategy has been established.

Radiotherapy for adrenal metastases has evolved considerably over time. Advances in treatment technology, particularly the development of intensity-modulated radiotherapy (IMRT), helical tomotherapy, and stereotactic body radiotherapy (SBRT), have enabled the delivery of ablative doses to adrenal targets while sparing adjacent critical organs [7]. Several retrospective studies have reported favorable local control (LC) rates and acceptable toxicity profiles with modern radiotherapy techniques for adrenal metastases from various primary tumors, including HCC [5,6,8,9]. More recently, magnetic resonance-guided radiotherapy (MRgRT) has emerged as a promising approach, enabling superior soft-tissue visualization and real-time tumor tracking, thereby further enhancing treatment precision [9,10].

In this context, we conducted a retrospective, bi-institutional study spanning two decades to evaluate tumor response, LC, survival outcomes, and treatment-related toxicity after radiotherapy for adrenal metastases from HCC.

## 2. Materials and Methods

### 2.1. Study Design and Patient Selection

This retrospective study was conducted at two tertiary referral institutions: Incheon St. Mary’s Hospital, The Catholic University of Korea (Institutional Review Board (IRB) No. OC24RIDI0127), and Inha University Hospital (IRB No. 2024-10-025). The medical records of patients who received radiotherapy for adrenal metastases from HCC between January 2005 and 31 December 2025 were reviewed. The data cut-off date for survival analysis was February 2026. The requirement for informed consent was waived because of the retrospective study design.

The inclusion criteria were as follows: (1) histologically confirmed HCC or a diagnosis established according to imaging criteria based on the American Association for the Study of Liver Diseases guidelines; (2) adrenal metastases confirmed on computed tomography (CT), magnetic resonance imaging (MRI), or positron emission tomography–computed tomography based on characteristic imaging features or interval growth in the setting of known advanced HCC; (3) radiotherapy administered to the adrenal metastases; and (4) availability of follow-up imaging for response assessment. Patients with a history of adrenal ablation or adrenal surgery were excluded. For the patient who underwent sequential bilateral adrenal radiotherapy, only the first treated lesion (left adrenal gland) was included in the analysis, and the contralateral adrenal recurrence was classified as a distant failure event. Among the 20 patients, 11 (55%) had additional extrahepatic metastases beyond the adrenal gland at the time of radiotherapy, 6 (30%) had no additional extrahepatic metastases, and 3 (15%) had uncertain extrahepatic status owing to limited staging information in early-era patients treated before the routine availability of positron emission tomography–computed tomography (PET/CT). A uniform definition of oligometastatic status could not be applied across the 20-year study period owing to heterogeneous staging methods and incomplete lesion-level documentation in early-era patients. Accordingly, this study was not restricted to oligometastatic patients. Adrenal radiotherapy was administered based on clinical judgment regarding local disease control and symptom management.

### 2.2. Radiotherapy

Radiotherapy was planned and delivered using various techniques according to institutional protocols and the available technology at the time of treatment, including three-dimensional conformal radiotherapy (3D-CRT), IMRT, or helical tomotherapy, and SBRT. Beginning in 2019, MRgRT was introduced at Incheon St. Mary’s Hospital using the MRIdian Linac system (ViewRay Inc., Oakwood Village, OH, USA). Practice variation was permitted based on tumor characteristics, institutional technology availability, and the proximity of the target to adjacent critical organs.

**Figure 1:**
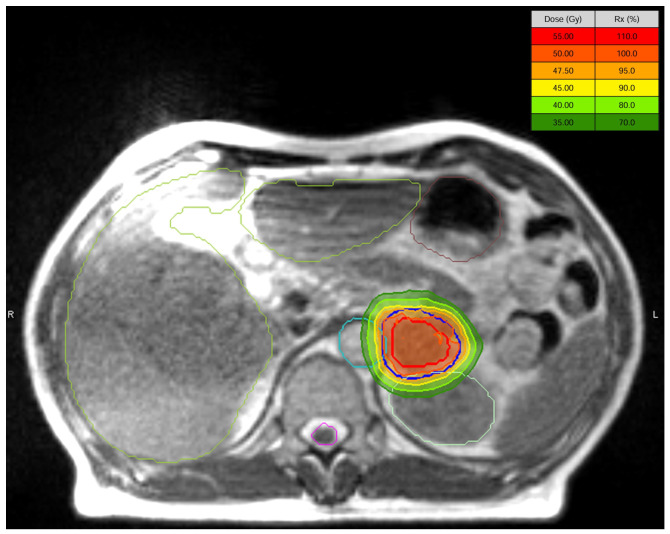
Axial magnetic resonance-guided radiotherapy (MRgRT) planning image of a left adrenal metastasis from hepatocellular carcinoma (50 Gy in 5 fractions; BED_10_, 100 Gy; MRIdian). The red contour indicates the gross tumor volume, and the blue contour indicates the planning target volume. Isodose lines are displayed in absolute dose: red line, 50 Gy (100%, prescription dose); orange line, 47.5 Gy (95%); yellow line, 45 Gy (90%); light green line, 40 Gy (80%); and green line, 35 Gy (70%). The remaining colored contours indicate organs at risk, including the spinal cord (magenta), stomach (brown), liver (olive green), left kidney (teal green), and great vessels (light blue). BED_10_, biologically effective dose with α/β = 10 Gy.

The gross tumor volume (GTV) was delineated based on contrast-enhanced CT and/or MRI simulation imaging. PET/CT was used for staging purposes in 3 patients, but was not used for target delineation in any patient. For cases treated with 3D-CRT or IMRT/helical tomotherapy (*n* = 12), a planning target volume (PTV) margin of 10 mm was applied to account for respiratory motion and setup uncertainty; immobilization was achieved using a vacuum cushion, and image guidance consisted of portal imaging in earlier-era cases and megavoltage CT or kV cone-beam computed tomography (CBCT) in later-era cases, according to available technology at the time of treatment. For SBRT cases (*n* = 2), four-dimensional CT simulation was performed with internal target volume-based planning and a 5 mm PTV expansion; CBCT-based image guidance was used. For MRgRT cases (*n* = 6), a 5 mm PTV margin was applied with real-time MR-based intrafraction motion monitoring and beam gating; further details of the MRgRT procedures have been previously described [11]. A representative treatment plan is shown in Figure 1. Dose constraints for organs at risk—including the liver, kidneys, stomach, duodenum, small intestine, large intestine, and spinal cord—were respected in accordance with institutional guidelines. The biologically effective dose (BED_10_) was calculated using the linear–quadratic model with an α/β ratio of 10 Gy. Concurrent or sequential systemic therapy administered during the study period was recorded, including the type and timing relative to radiotherapy.

### 2.3. Response Evaluation and Statistical Analysis

Tumor response of the adrenal metastases was assessed according to the Response Evaluation Criteria in Solid Tumors (RECIST) version 1.1 based on changes in the longest diameter on CT and/or MRI. Follow-up imaging was performed at the discretion of the treating physician, typically every 2–3 months after radiotherapy, and the time point of maximal tumor size reduction was used to define the best response. RECIST 1.1 criteria based on CT and/or MRI were applied uniformly for response assessment throughout the study period. PET/CT was not used for response assessment in any patient. Response categories were defined as complete response (CR), partial response (PR), stable disease (SD), and progressive disease (PD). The objective response rate (ORR) was defined as the proportion of patients who achieved CR or PR, and the disease control rate (DCR) was defined as the proportion who achieved CR, PR, or SD.

Adrenal LC, OS, and progression-free survival (PFS) were estimated using the Kaplan–Meier method. LC was defined as the absence of radiologic progression at the irradiated site, OS as the time from radiotherapy initiation to death or last follow-up, and PFS as the time to radiologic progression at any site or death. Adrenal local recurrence (LR) was defined as documented progression at the irradiated site according to RECIST version 1.1. The association between BED_10_ (dichotomized as ≥75 vs. <75 Gy) and adrenal LR was assessed using Fisher’s exact test. A post hoc exploratory analysis was performed using a BED_10_ threshold of 75 Gy. No formal cutoff optimization was performed. Univariate and multivariable analyses for prognostic factors were not performed because of the limited sample size. Toxicity was graded according to the Common Terminology Criteria for Adverse Events version 5.0. All analyses were performed using IBM SPSS Statistics (version 28.0; IBM Corp., Armonk, NY, USA) and R (version 4.3.0; R Foundation for Statistical Computing, Vienna, Austria). A two-sided *p* < 0.05 was considered statistically significant.

## 3. Results

### 3.1. Patient and Treatment Characteristics

A total of 20 patients with adrenal metastases from HCC who received radiotherapy between January 2005 and December 2025 were included in this study. The clinical and treatment characteristics are summarized in Table 1 and Table 2, and the individual treatment courses are illustrated in Supplementary Figure S1. Briefly, the median age was 63 years (range, 40–83); 17 patients (85%) were male. Most patients had Child–Pugh class A liver function (85%) and ECOG performance status 0 or 1. Intrahepatic HCC was uncontrolled in 50% at the time of radiotherapy, and portal vein tumor thrombosis (PVTT) was present in 55%. Adrenal metastases were metachronous in 16 patients (80%), with a median interval from HCC diagnosis of 8.4 months.

Regarding radiotherapy, the median total dose was 47.5 Gy (range, 25–60 Gy), delivered in a median of 10 fractions (range, 4–25), with a median fraction size of 3.33 Gy (range, 1.8–10 Gy). The median BED_10_ was 63.3 Gy (range, 31.2–100.0 Gy). The radiation techniques used were 3D-CRT in four patients (20%), IMRT or helical tomotherapy in eight patients (40%), SBRT in two patients (10%), and MRgRT in six patients (30%). The median GTV and PTV were 78.5 mL (*n* = 18) and 153.2 mL (*n* = 19), respectively. GTV data were unavailable for two patients treated in the early era, whose planning records were accessible only as scanned documents, and PTV data were unavailable for one patient for the same reason.

Systemic therapy was administered in 13 patients (65%), including chemotherapy in 5 (25%), TKIs in 3 (15%), and ICIs in 5 (25%; atezolizumab plus bevacizumab in 4 and nivolumab in 1). Seven patients (35%) did not receive systemic therapy at any time relative to radiotherapy. With respect to timing, systemic therapy was administered before radiotherapy in 5 patients, concurrently in 8 patients, and after radiotherapy in 10 patients; the total exceeds 13 because some patients received systemic therapy during multiple time periods. In addition, concurrent intrahepatic transarterial chemoembolization (TACE) was performed in seven patients (35%) around the time of adrenal radiotherapy to target primary liver lesions.

### 3.2. Tumor Response

Tumor response was evaluable in all 20 patients. No patient achieved a CR. The absence of complete responses on imaging is consistent with the broader radiotherapy literature for adrenal metastases and may reflect persistent fibrotic or necrotic tissue despite local tumor control. A PR was observed in 11 patients (55.0%), SD in 8 (40.0%), and PD in 1 (5.0%). The ORR was 55.0%, and the DCR was 95.0%. Among patients who achieved an objective response (*n* = 11), the median time from radiotherapy initiation to the best response was 2.8 months (range, 1.5–9.3 months).

### 3.3. Adrenal Local Control

**Figure 2:**
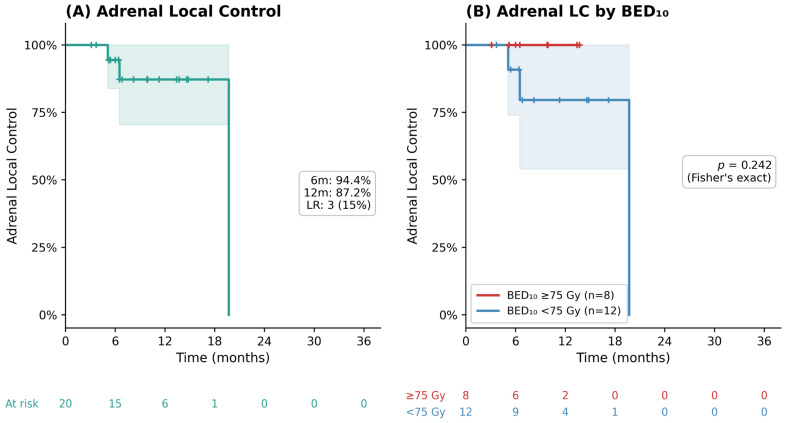
(**A**) Adrenal local control (LC; 6-/12-month rates: 94.4%/87.2%; 3 local recurrences, 15.0%) and (**B**) Adrenal LC stratified by BED_10_ (≥75 vs. <75 Gy): no local recurrence was observed at BED_10_ ≥ 75 Gy (0/8, 100% LC) compared with 25.0% (3/12) at BED_10_ < 75 Gy (*p* = 0.242, Fisher’s exact test). Tick marks indicate censored observations. Numbers at risk are shown below each panel. BED_10_, biologically effective dose with α/β = 10 Gy.

Adrenal LR was observed in three patients (15.0%), each demonstrating PD at the irradiated adrenal site on follow-up imaging. The 6-month and 12-month adrenal LC rates were 94.4% and 87.2%, respectively (Figure 2A). The Kaplan–Meier-estimated 12-month LC rate of 87.2% reflects censored observations and therefore differs from the crude LR rate of 15.0% (3/20).

When stratified by BED_10_, no adrenal LR was observed among patients who received BED_10_ ≥ 75 Gy (0/8, 100% LC), whereas 3 of 12 patients (25.0%) in the BED_10_ < 75 Gy group developed adrenal LR (*p* = 0.242, Fisher’s exact test) (Figure 2B). This difference did not reach statistical significance and should be interpreted as exploratory and hypothesis-generating, given the limited sample size.

### 3.4. Disease Failure Pattern

Disease failure was observed in 13 of 20 patients (65.0%) during follow-up (Table 3). Among these patients, isolated local (adrenal) failure occurred in 1 patient (7.7%), isolated intrahepatic failure in 2 (15.4%), isolated extrahepatic failure in 6 (46.2%), and multiple-site failure in 4 (30.8%); 2 of the latter also experienced adrenal progression, accounting for all 3 patients (15.0%) with adrenal LR. The most common failure pattern was extrahepatic progression outside the irradiated adrenal gland.

### 3.5. Survival Outcomes

**Figure 3:**
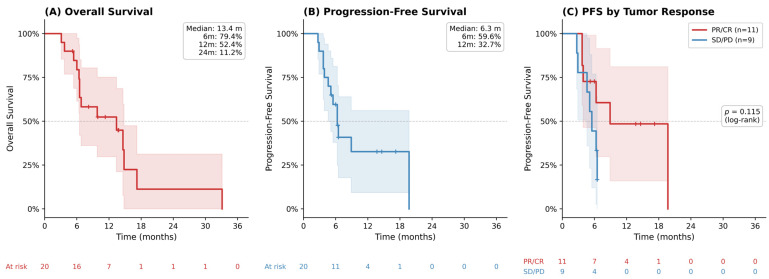
Kaplan–Meier curves for (**A**) overall survival (OS; median 13.4 months; 6-/12-/24-month rates: 79.4%/52.4%/11.2%), (**B**) progression-free survival (PFS; median 6.3 months; 6-/12-month rates: 59.6%/32.7%), and (**C**) PFS stratified by tumor response (partial or complete response vs. stable or progressive disease; *p* = 0.115, log-rank test). Tick marks indicate censored observations. Numbers at risk are shown below each panel. OS, overall survival; PFS, progression-free survival; PR, partial response; SD, stable disease; PD, progressive disease.

The median follow-up duration was 9.0 months (range, 3.1–33.1 months). At the time of analysis, 14 patients had died, and 6 were alive. The median OS was 13.4 months, with 6-, 12-, and 24-month survival rates of 79.4%, 52.4%, and 11.2%, respectively (Figure 3A). Radiologic disease progression was documented in 13 patients (65.0%), resulting in a median PFS of 6.3 months; the 6- and 12-month PFS rates were 59.6% and 32.7%, respectively (Figure 3B).

Among the five patients who received ICI-based systemic therapy (atezolizumab plus bevacizumab, *n* = 4; nivolumab, *n* = 1), ICI was administered concurrently with radiotherapy in three patients and sequentially after radiotherapy in two patients. Adrenal local recurrence was observed in two of the five ICI-treated patients (40.0%). Among the five ICI-treated patients, two died, and three remained alive with disease at last follow-up (median follow-up, 11.3 months; range, 9.8–13.7 months). Given the small number of patients, these data are purely descriptive, and no conclusions regarding the interaction between radiotherapy and ICI can be drawn.

### 3.6. Treatment-Related Toxicity

Treatment-related toxicities are summarized in Table 4. Three patients (15%) experienced no treatment-related toxicity. Regarding radiotherapy-related acute toxicities, the most commonly observed constitutional symptom was fatigue, occurring in three patients (15%). Gastrointestinal symptoms were observed in seven patients (35%), including anorexia in two (10%), nausea in two (10%), diarrhea in two (10%), and vomiting in two (10%). All radiotherapy-related toxicities were grade 1 or 2, and no grade ≥ 3 radiotherapy-related toxicity was observed.

Hematologic toxicities were observed in 12 patients (60%), including leukopenia in 11 (55%), anemia in 9 (45%), and thrombocytopenia in 8 (40%). Grade ≥ 3 hematologic toxicity occurred in seven patients (35%), including leukopenia in five and thrombocytopenia in four patients (with overlap). These events were largely attributed to underlying hepatic dysfunction, hypersplenism, and/or concurrent systemic therapy rather than to direct radiation effects.

With respect to late toxicity, grade 1 gastrointestinal bleeding was observed in 1 patient (5%) and resolved with conservative management. No grade ≥ 2 late toxicity was documented. No radiation-induced renal or spinal cord injuries were observed. No clinically overt adrenal insufficiency requiring hormonal intervention was documented during the study period; however, biochemical adrenal function was not systematically assessed, and subclinical insufficiency cannot be excluded.

## 4. Discussion

Despite the recognized clinical significance of adrenal metastases from HCC as an important target for local ablative therapy, prospective data on the efficacy of radiotherapy in this specific setting remain limited, and most prior series predominantly reflect the conventional fractionated radiotherapy era. To our knowledge, the present series represents one of the longest-running HCC-specific radiotherapy series for adrenal metastases reported to date, spanning two decades of technological evolution from 3D-CRT to MRgRT. Although our cohort is substantially smaller than Korean Radiation Oncology Group (KROG) 13-05 (*n* = 134) [5] and Yuan et al. (*n* = 81) [6], our findings are intended to be complementary rather than competing evidence, and all results should be interpreted as hypothesis-generating given the limited sample size. In this 20-year, bi-institutional retrospective study, radiotherapy achieved an ORR of 55.0%, a 12-month adrenal LC rate of 87.2%, and a median OS of 13.4 months.

The ORR of 55.0% compares favorably with the 38.3% reported by Jung et al. (KROG 13-05, *n* = 134, 88% treated with 3D-CRT) [5] and is comparable to the 55.6% reported by Yuan et al. with helical tomotherapy (*n* = 81) [6]. In the SBRT era, Cheng et al. reported an ORR of 86.0% among 52 patients with HCC treated with CyberKnife-based SBRT and concurrent systemic therapy [12]; this higher ORR likely reflects the predominance of SBRT and the greater proportion of patients receiving systemic therapy in that cohort. The absence of complete responses is consistent with findings from the broader radiotherapy literature on adrenal metastases and may reflect persistent fibrotic or necrotic tissue after tumor regression. Regarding adrenal LC, our 12-month LC rate of 87.2% compares favorably with the 1-year LC rate of 77.8% reported by Xu et al. [13] and falls within the actuarial 1-year LC range of 55–97% reported across broader series, including Franzese et al. (*n* = 142, 1-year LC 85.4%) [14] and Facondo et al. (*n* = 24, ORR 66.5%) [15], as well as multiple non-HCC adrenal SBRT series [7,16,17,18,19,20]. In contrast, most prior HCC-specific series were conducted predominantly in the conventional radiotherapy era and included relatively small sample sizes [5,6,8], underscoring the need for updated evidence encompassing modern radiotherapy techniques and the current systemic therapy landscape.

The median OS of 13.4 months is consistent with these prior HCC-specific series (10–15 months) [5,6]. Cheng et al. reported a higher median OS of 22 months [12], likely reflecting the higher rates of systemic therapy in their cohort (ICI 42.1%, TKI 68.3%) and better intrahepatic disease control. In contrast, 50% of our patients had uncontrolled intrahepatic disease, and only 25% received ICI; PVTT was present in 55%, indicating a predominantly Barcelona Clinic Liver Cancer stage C population. The inclusion of patients treated before the availability of ICI-based regimens likely attenuated the overall OS estimate. These differences in patient selection and treatment context, rather than differences in radiotherapy technique per se, likely account for the survival differences across studies.

Disease failure was predominantly driven by systemic progression rather than by local failure. Among the 13 patients with documented disease failure, extrahepatic progression outside the irradiated adrenal gland was the most common pattern (six patients, 46.2%), followed by multiple-site failure (four patients, 30.8%); isolated adrenal failure occurred in only one patient (7.7%). This pattern mirrors findings from the broader radiotherapy literature on adrenal metastases, in which out-of-field systemic progression is the dominant mode of failure [7]. Among the 14 patients who died, systemic disease progression was the predominant cause of death. These findings underscore the need to combine effective local radiotherapy with active systemic therapy to improve overall outcomes in this population.

Regarding treatment-related toxicity, all radiotherapy-related toxicities in our cohort were grade 1 or 2, with no grade ≥ 3 events observed despite the inclusion of both conventional and modern radiotherapy techniques. This favorable toxicity profile is consistent with findings from HCC-specific SBRT series: Cheng et al. [12] reported no grade ≥2 radiotherapy-related adverse events among 52 patients, and Xu et al. [13] reported only one case of grade 3 hepatic injury likely attributable to concurrent targeted therapy rather than radiotherapy alone. In the broader adrenal metastasis literature, Franzese et al. [14] reported grade ≥ 3 toxicity in 0.7% of patients, and Facondo et al. [15] reported acute toxicity in 48% of patients, predominantly grade 1–2, confirming the general safety of modern ablative radiotherapy for adrenal metastases.

In an exploratory post hoc analysis, no adrenal LR occurred among patients receiving BED_10_ ≥ 75 Gy (0/8, 100% LC), compared with an LR rate of 25.0% (3/12) in the BED_10_ < 75 Gy group (*p* = 0.242, Fisher’s exact test). This finding is consistent with the prior literature. A 2022 systematic review identified BED as the most consistent predictor of LC, with no local failures observed at BED_10_ > 100 Gy [7]. Arcidiacono et al. identified BED_10_ ≥ 72 Gy as a threshold for improved LC in adrenal SBRT (*p* = 0.05) [21]. In addition, a multi-institutional magnetic resonance-guided SBRT analysis of 269 adrenal metastases reported no LR at BED_10_ > 100 Gy [22]. The numerically shorter OS observed in the high-BED10 group (9.8 vs. 14.6 months, *p* = 0.338) is most likely attributable to era-related confounding: the short median follow-up of 9.0 months relative to the 20-year accrual period reflects the predominance of recently treated patients in the high-BED group, who had less time to accumulate follow-up. This comparison should not be interpreted causally.

This study documents the 20-year evolution of radiotherapy technology for adrenal metastases across two Korean institutions. Image guidance evolved from X-ray-based portal imaging and megavoltage computed tomography-guided helical tomotherapy to cone-beam computed tomography-guided SBRT and MRgRT at one institution beginning in 2019, enabling real-time magnetic resonance (MR)-based tumor tracking [22], whereas the other institution employed linear accelerator (linac)-based 3D-CRT and IMRT throughout. The apparent consistency of LC outcomes across technique eras in this small cohort is an observation of interest. However, given the small numbers within each subgroup and the confounding effects of dose, era, and patient selection, no conclusions regarding the relative contributions of technique or image-guidance modality on adrenal LC can be drawn. Larger prospective comparisons are required to evaluate whether technique selection or image-guidance modality independently influences adrenal LC. Clinical outcomes of MRgRT for adrenal metastases have been reported in multiple series [9,10,20,23,24,25]. The study period also spans a fundamental shift in systemic therapy for HCC. The adrenal gland has been postulated to represent an immune-privileged sanctuary site [26], and the combination of radiotherapy and ICIs has been hypothesized to enhance antitumor immunity. However, the present data (ICI *n* = 5) are insufficient to support conclusions regarding radiotherapy–ICI interactions, and a dedicated prospective investigation is warranted.

Several limitations warrant consideration. The retrospective design and small sample size (*n* = 20) limit statistical power and preclude multivariable analysis; all findings should be considered hypothesis-generating and require validation in larger, prospective series. The BED10 threshold of 75 Gy was not pre-specified; this analysis should be interpreted as exploratory only, and no formal cutoff optimization was performed. Multiple exploratory comparisons were not adjusted for type I error. The short median follow-up of 9.0 months relative to the 20-year accrual period reflects the predominance of recently treated patients, which introduces era-related confounding; in particular, survival comparisons between subgroups should not be interpreted causally. Heterogeneity of techniques across two decades introduces additional treatment-era confounding. The predominantly hepatitis B virus-associated Korean population may limit generalizability. Adrenal function was not systematically assessed. Although no clinically overt adrenal insufficiency requiring hormonal replacement was documented, the bilateral case warrants particular attention, and subclinical insufficiency cannot be excluded. Concurrent TACE in 35% of patients, combined with underlying liver dysfunction, may have contributed to hematologic toxicity. Notwithstanding these limitations, the bi-institutional design and two-decade span provide real-world data supporting prospective studies integrating precision radiotherapy with systemic therapies.

## 5. Conclusions

In this 20-year, bi-institutional retrospective study, radiotherapy demonstrated consistent local control and acceptable toxicity for adrenal metastases from HCC across the full spectrum of technological evolution from 3D-CRT to MRgRT. Given the limited sample size and retrospective design, these findings should be considered hypothesis-generating and require prospective validation. Prospective studies integrating precision radiotherapy with immune checkpoint inhibitor-based regimens are warranted to define the optimal multimodal strategy for this population.

## Figures and Tables

**Table 1:** Patient characteristics (N = 20).

Variable		No. (%) or Median (Range)
Age at radiotherapy (years)	Median (range)	63 (40–83)
Sex		
Male		17 (85%)
Female		3 (15%)
ECOG performance status		
0		6 (30%)
1		14 (70%)
HCC etiology		
Hepatitis B virus		13 (65%)
Hepatitis C virus		2 (10%)
Alcoholic		3 (15%)
Others		2 (10%)
Liver cirrhosis	Yes	7 (35%)
Child–Pugh class		
A		17 (85%)
B		3 (15%)
Intrahepatic HCC status at RT		
Controlled		10 (50%)
Uncontrolled		10 (50%)
Number of intrahepatic tumors		
Solitary		4 (20%)
Multiple (≥2)		15 (75%)
No viable tumor		1 (5%)
Portal vein tumor thrombosis	Yes	11 (55%)
Additional extrahepatic metastases		
Yes ^a^		11 (55%)
Lung		5
Bone		5
Lymph node		2
Brain		1
Peritoneum		1
No		6 (30%)
Unknown		3 (15%)
Timing of adrenal metastasis		
Synchronous		4 (20%)
Metachronous		16 (80%)
Interval, HCC diagnosis to adrenal metastasis (months)	Median (range)	8.4 (0–94.1)
Interval, adrenal diagnosis to RT (months)	Median (range)	1.8 (0.3–8.9)
Adrenal tumor location		
Right		7 (35%)
Left		12 (60%)
Bilateral		1 (5%)
Adrenal tumor size (cm)	Median (range)	5.5 (1.4–8.6)
Serum AFP (ng/mL)	Median (range)	321.7 (1.2–494,717)

^a^ Overlap permitted. The number of involved organ sites per patient ranged from 1 to 4. Abbreviations: RT, radiotherapy; ECOG, Eastern Cooperative Oncology Group; HCC, hepatocellular carcinoma; AFP, alpha-fetoprotein.

**Table 2:** Radiotherapy and treatment characteristics (N = 20).

Variable		No. (%) or Median (Range)
Total radiation dose (Gy)	Median (range)	47.5 (25–60)
Dose per fraction (Gy)	Median (range)	3.33 (1.8–10)
Number of fractions	Median (range)	10 (4–25)
BED_10_ (Gy_10_)	Median (range)	63.3 (31.2–100.0)
≥75 Gy_10_		8 (40%)
Radiation technique		
3D-CRT	4 (20%)	1.8 Gy × 25 fx − 3; 2.5 Gy × 10 fx − 1
IMRT or Helical tomotherapy	8 (40%)	3.0–3.33 Gy/fx, 10–25 fx
SBRT	2 (10%)	8 Gy × 4 fx − 1; 10 Gy × 4 fx − 1
MRgRT (MRIdian)	6 (30%)	5–10 Gy/fx, 4–10 fx
GTV (mL) ^a^	Median (range)	78.5 (3.7–437.1)
PTV (mL) ^b^	Median (range)	153.2 (13.8–647.9)
Concurrent systemic therapy		
None		5 (25%)
TACE		7 (35%)
ICI		3 (15%)
TKI		3 (15%)
Chemotherapy		2 (10%)
Any systemic therapy ^c^		
ICI		5 (25%)
TKI		3 (15%)
Chemotherapy		5 (25%)
None		7 (35%)

^a^ GTV data available for 18 patients; ^b^ PTV data available for 19 patients; ^c^ Includes pre-, concurrent, and post-RT therapy with overlap permitted. Abbreviations: BED_10_, biologically effective dose (α/β = 10 Gy); 3D-CRT, three-dimensional conformal radiotherapy; IMRT, intensity-modulated radiotherapy; SBRT, stereotactic body radiotherapy; MRgRT, MR-guided radiotherapy; GTV, gross tumor volume; PTV, planning target volume; TACE, transarterial chemoembolization; ICI, immune checkpoint inhibitor; TKI, tyrosine kinase inhibitor.

**Table 3:** Patterns of disease failure (N = 20).

Failure Pattern	N (%)	Sites of Failure
No failure	7 (35.0%)	—
Local only	1 (7.7%) ^a^	Irradiated adrenal gland
Intrahepatic only	2 (15.4%)	Intrahepatic HCC progression
Extrahepatic only ^b^	6 (46.2%)	Lung (n = 2), Bone (n = 2), Lung + Bone (n = 1), Lung + LN + Brain (n = 1)
Multiple sites	4 (30.8%)	Liver + Bone (n = 1), Liver + Adrenal (n = 1), Lung + Adrenal (n = 1), Liver + Peritoneum (n = 1)
Total failure	13 (65.0%)	

^a^ Percentages for failure patterns are calculated among the 13 patients with disease failure. ^b^ Excludes the irradiated adrenal site. Abbreviations: LN, lymph node.

**Table 4:** Treatment-related toxicities (CTCAE v5.0, N = 20).

Toxicity (CTCAE v5.0)	Grade 1 N (%)	Grade 2 N (%)	Grade ≥ 3 N (%)	Any Grade N (%)
Acute toxicity—RT-related				
Fatigue	3 (15%)	0	0	3 (15%)
Nausea	2 (10%)	0	0	2 (10%)
Vomiting	1 (5%)	1 (5%)	0	2 (10%)
Anorexia	0	2 (10%)	0	2 (10%)
Diarrhea	2 (10%)	0	0	2 (10%)
Acute toxicity—Hematologic ^a^				
Leukopenia	1 (5%)	5 (25%)	5 (25%)	11 (55%)
Thrombocytopenia	1 (5%)	3 (15%)	4 (20%)	8 (40%)
Anemia	1 (5%)	6 (30%)	2 (10%)	9 (45%)
Late toxicity				
GI bleeding	1 (5%)	0	0	1 (5%)

^a^ Hematologic toxicities were presumed to be largely attributable to underlying liver dysfunction and hypersplenism related to hepatic cirrhosis and/or concurrent systemic therapy, rather than to direct radiation effects, though a contribution from radiotherapy cannot be entirely excluded. Abbreviations: CTCAE, Common Terminology Criteria for Adverse Events; GI, gastrointestinal; RT, radiotherapy.

## Data Availability

The datasets generated during and/or analyzed during the current study are not publicly available due to patient privacy, but are available from the corresponding author on reasonable request.

## Supplementary Materials

The following supporting information can be downloaded at: https://www.mdpi.com/article/10.3390/curroncol33060328/s1. Figure S1: Swimmer plot of individual treatment courses and clinical outcomes (N = 20).

## Abbreviations

The following abbreviations are used in this manuscript:

3D-CRT	three-dimensional conformal radiotherapy
AFP	alpha-fetoprotein
BED_10_	biologically effective dose with α/β = 10 Gy
CBCT	cone-beam computed tomography
CR	complete response
CT	computed tomography
CTCAE	Common Terminology Criteria for Adverse Events
DCR	disease control rate
ECOG	Eastern Cooperative Oncology Group
GI	gastrointestinal
GTV	gross tumor volume
HCC	hepatocellular carcinoma
ICI	immune checkpoint inhibitor
IMRT	intensity-modulated radiotherapy
IRB	Institutional Review Board
KROG	Korean Radiation Oncology Group
LC	local control
LN	lymph node
LR	local recurrence
MRI	magnetic resonance imaging
MRgRT	MR-guided radiotherapy
ORR	objective response rate
OS	overall survival
PET	positron emission tomography
PD	progressive disease
PFS	progression-free survival
PR	partial response
PTV	planning target volume
PVTT	portal vein tumor thrombosis
RECIST	Response Evaluation Criteria in Solid Tumors
RT	radiotherapy
SBRT	stereotactic body radiotherapy
SD	stable disease
TACE	transarterial chemoembolization
TKI	tyrosine kinase inhibitor
