# Supplementary figures and images for: Radiotherapy for Adrenal Metastases from Hepatocellular Carcinoma: A 20-Year Bi-Institutional Experience
Source: Curr Oncol. 2026 Jun 1;33(6):328. doi: 10.3390/curroncol33060328 (PMC13297966; doi:10.3390/curroncol33060328)

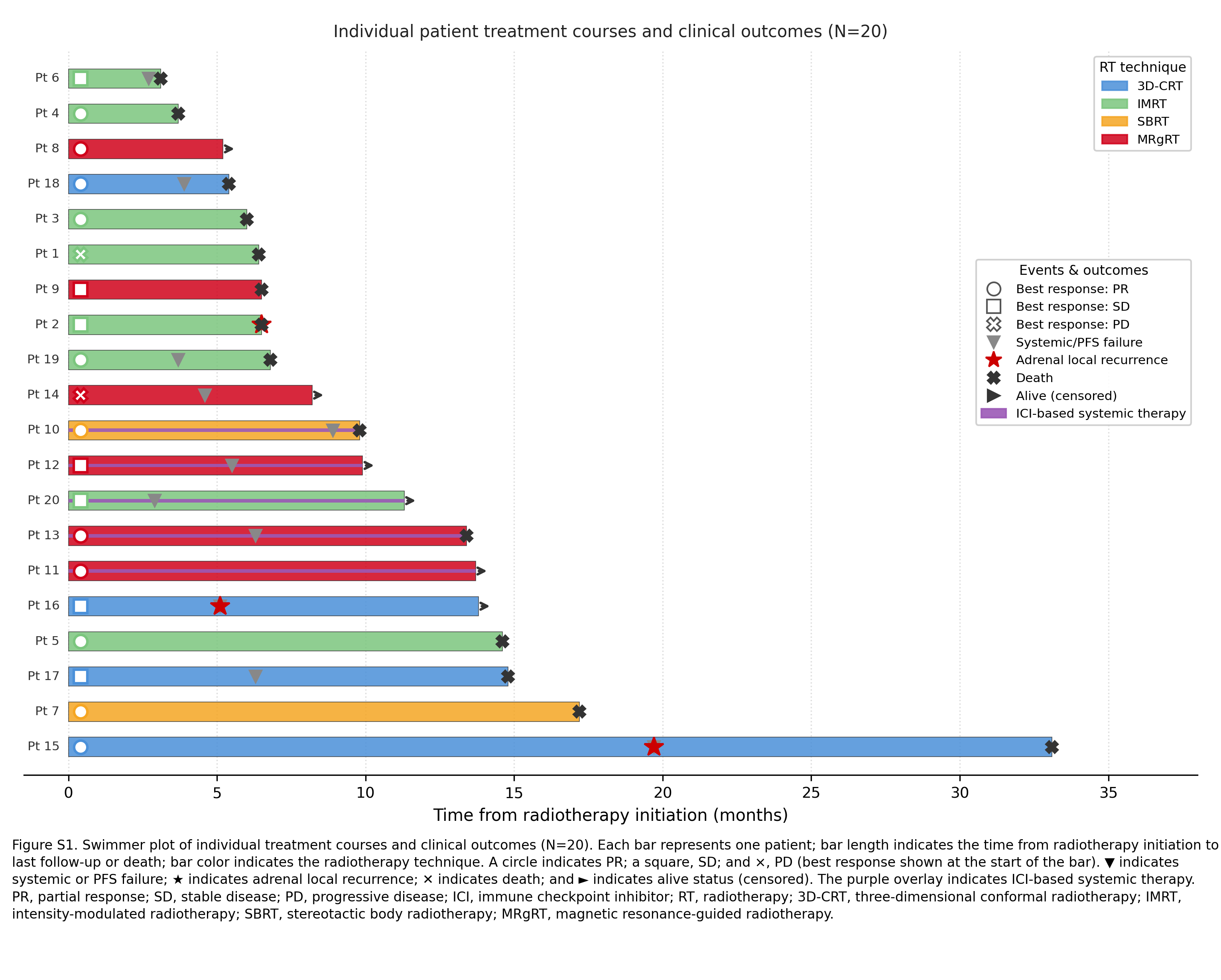

Supplement: Supplementary file 1 [file curroncol-33-00328-s001.zip › SuppFig1_with_caption.tiff]
